# Cytokine-associated neutrophil extracellular traps and antinuclear antibodies in *Plasmodium falciparum *infected children under six years of age

**DOI:** 10.1186/1475-2875-7-41

**Published:** 2008-02-29

**Authors:** Virginia S Baker, Godwin E Imade, Norman B Molta, Pallavi Tawde, Sunday D Pam, Michael O Obadofin, Soloman A Sagay, Daniel Z Egah, Daniel Iya, Bangmboye B Afolabi, Murray Baker, Karen Ford, Robert Ford, Kenneth H Roux, Thomas CS Keller

**Affiliations:** 1 Department of Biological Science, Florida State University, Tallahassee, Florida, USA; 2 Jos University Teaching Hospital and Medical School, Jos, Nigeria; 3 Jos University, Jos, Nigeria; 4 Nigerian Institute of Medical Research, Yaba, Nigeria; 5 Jackson Hospital, Marianna, FL, USA; 6 World Health Mission, Pittsburgh, Pennsylvania, USA; 7 Chipola College, Marianna, FL 32446, USA

## Abstract

**Background:**

In *Plasmodium falciparum*-infected children, the relationships between blood cell histopathology, blood plasma components, development of immunocompetence and disease severity remain poorly understood. Blood from Nigerian children with uncomplicated malaria was analysed to gain insight into these relationships. This investigation presents evidence for circulating neutrophil extracellular traps (NETs) and antinuclear IgG antibodies (ANA). The presence of NETs and ANA to double-stranded DNA along with the cytokine profiles found suggests autoimmune mechanisms that could produce pathogenesis in children, but immunoprotection in adults.

**Methods:**

Peripheral blood smear slides and blood samples obtained from 21 Nigerian children under six years of age, presenting with uncomplicated malaria before and seven days after initiation of sulphadoxine-pyrimethamine (SP) treatment were analysed. The slides were stained with Giemsa and with DAPI. Levels of the pro-inflammatory cytokines IFN-γ, IL-2, TNF, CRP, and IL-6, select anti-inflammatory cytokines TGF-β and IL-10, and ANA were determined by immunoassay.

**Results:**

The children exhibited circulating NETs with adherent parasites and erythrocytes, elevated ANA levels, a Th2 dominated cytokine profile, and left-shifted leukocyte differential counts. Nonspecific ANA levels were significant in 86% of the children pretreatment and in 100% of the children seven days after SP treatment, but in only 33% of age-matched control samples collected during the season of low parasite transmission. Levels of ANA specific for dsDNA were significant in 81% of the children both pre-treatment and post treatment.

**Conclusion:**

The results of this investigation suggest that NET formation and ANA to dsDNA may induce pathology in falciparum-infected children, but activate a protective mechanism against falciparum malaria in adults. The significance of in vivo circulating chromatin in NETs and dsDNA ANA as a causative factor in the hyporesponsiveness of CpG oligonucleotide-based malaria vaccines is discussed.

## Background

Pathogenesis in humans infected with *Plasmodium falciparum *involves a complex multifactorial immune system response to the parasite as well as to host cell and tissue damage. Although much is known about the immunological response to falciparum infection [1-3], relationships between immunocompetence [4] and disease severity remain poorly understood. Patient age [5], genetics [6], vitamin sufficiency [7-10], gravidae [11-13], control of oxidative stress [14-16], and factors related to the availability of complement proteins and their receptors [17-23] all affect immunocompetence, as does the presence of immunosuppressive [4] and autoimmune factors [24].

The levels of certain cytokines associated with falciparum malaria can provide clues to the immune system reaction, but analyses of cytokine levels alone can yield paradoxical results concerning the protection and pathology of the underlying highly integrated responses [5,15,18,25-35]. An IFN-γ-Th1-dependent immune response in the mouse model, for example, has been associated with both immunoprotection [3] and immunopathology [36]. Likewise, elevated CRP levels can both activate the classical complement cascade and yet provide protection for endothelial cells from membrane attack complex deposition through up regulation of surface receptor expression to counter the effects of the activated cascade [23].

The immune response to falciparum infection may depend not only on the cytokine profile but also on hematologic activity. Recently, a novel activity of neutrophils, formation of neutrophil extracellular traps (NETs), has been described [37-42]. NETs can bind and kill a variety of microbes [38,42], but NET formation has not been described previously as a response to falciparum malaria infection.

The goal of this study was to investigate the cytokine profiles and hematologic activity involved in the immune response to falciparum infection in children six years of age or younger presenting with uncomplicated malaria. Samples from 21 falciparum-infected children from a mesoendemic region of Nigeria were analysed by ELISA for levels of IL-10, IL-6, IL-2, TNF, IFN-γ, TGF-β, CRP, and circulating antinuclear antibodies (ANA) and by peripheral slide analysis for leukocyte differential count and the presence of abnormally fragile leukocytes and NETs. The results of this investigation suggest mechanisms by which immunoprotection develops, as well as mechanisms by which autoimmune activity initially may lead to increased pathogenesis in children, but over time may induce immunoprotection in the exposed adult population. Evidence for NET formation and autoimmune activity also suggests a mechanism by which the presence of ANA to dsDNA may contribute to a hyporesponsiveness to CpG-based malarial vaccines [4,43,44].

## Methods

### Study site

Jos University Teaching Hospital (JUTH) and Florida State University Human Subjects Committees approved the protocol for a physician-based malaria team from Jos, Nigeria, to perform a malaria clinical outreach/study of children under six years of age in the Barkin Ladi Village Clinic [45]. The Barkin Ladi Village Clinic is located in a region mesoendemic to *P. falciparum *infection with average spleen rate of 28-30% during high transmission season and hyperparasitaemia occurring in 0.6% of the patients [46] and represents the situation found in many parts of sub-Saharan Africa, where advanced hospital facilities are not available to the patient, physician, or researcher.

### Patients and sampling

Patients presenting with slide positive falciparum malaria were evaluated by a physician for general signs of neurological well-being, hepatomegaly, splenomegaly, fever, and wasting. All patients in the study were characterized as having uncomplicated falciparum malaria through established criteria [26,46,47]. Patients were given a single therapeutic dose of sulphadoxine-pyrimethamine (SP, *Fansidar^®^*), previously assessed by members of the current research team as the drug of choice for the Plateau region of Nigeria because of minimal trophozoite recrudescence [46]. Patients placed on the study received a seven-day post-treatment follow-up visit by the physicians. All patients were given an insecticide treated mosquito net to reduce the rate of re-infection whether they participated in the study or not.

Blood from slide-positive children under six years of age that was sero-negative for HIV (Oraquick Rapid Antibody HIV Test #3001-0951, OraSure Technologies Inc, Bethlehem, PA), and that had a packed cell volume greater than 25% [47] was collected pre- and seven-days post SP-treatment. Blood samples were collected in sterile blood collecting vacutainers with 15% EDTA, 3.8% Na^+ ^citrate, or without additive for serum collection (Becton Dickinson, Franklin Lakes, N.J.). The EDTA-containing samples were immediately placed on ice. The Na^+ ^citrate and serum samples were placed in a foam-insulated cooler with an ice pack tethered inside to maintain a cool environment without contact of the tubes with the coolant. Blood samples were taken at the end of the day so that all samples collected were centrifuged within an hour and a half of collection. Na^+ ^citrate and serum samples were spun at ambient temperature (27°C) for 15 minutes at 1000 × g, whereas EDTA samples were spun at 4°C for 15 minutes at 1000 × g in a refrigerator centrifuge on site (powered with a generator). The centrifuge had adequate bucket space for simultaneous spinning of all serum and Na^+ ^citrate samples at ambient temperature first, followed by centrifugation of EDTA samples at 4°C. Retrieved serum and plasma samples were transferred aseptically to Nalgene cryovials (Fisher Scientific) and stored at -70°C in a freezer at JUTH. Samples were transported by carrier in a Saf T Case (STP 350, SAF-T-PAK, Inc) with all enclosed samples, containers, and gel packs (Arctic Pack, Packaging Products Corp., New Bedford, Mass) frozen at -70°C before air transport to FSU for analysis.

### Histology

Films of freshly collected finger-prick blood samples were stained in Giemsa solution diluted in pH 7.3 phosphate buffer. A rapid staining protocol using double strength Giemsa (4%) for 10 minutes was used initially during screening to select those who had malaria parasitaemia. For parasite density estimation, a second slide was stained with 2% Giemsa solution for 30 minutes. In both screening and subsequent parasite density estimation, the slides were fixed with absolute methanol and allowed to air dry before staining [48]. Two trained microscopists evaluated the slides for the presence of falciparum malaria. Peripheral blood smears from patients present at the clinic, but slide negative for falciparum malaria, were used as staining controls. A leukocyte differential analysis of slides shipped to the US was performed by two microscopists, one of whom was a physician with expertise in abnormal leukocyte fragility, detection of NETs dispersed among normal leukocytes, and the identification of neutrophil toxic granulation. The pre-treatment differential between metamyelocytes, segmented neutrophils, bands, hypersegmented neutrophils, eosinophils, basophils, lymphocytes, monocytes, NETs, and smudge forms was calculated per 100 leukocytes for each child studied pre treatment (n = 21) and post treatment (n = 19 – two slides were unavailable for differential count in U.S.). The slides were scored for Rouleaux and coined-agglutinin aggregation and for the presence or absence of NET-associated extracellular fibers sequestering parasites.

To determine the presence of chromatin in NET formation, slides were destained by the protocol of Kobayashi et al, 1989 [49]. Briefly, the slides were immersed in 50% ethanol at 37°C for one hour and 100% methanol at 37°C for one hour. The slides were then stained with DAPI (0.01 mg/ml in Tris-EDTA buffer solution with 10 mM 2-mercaptoethylamine, pH 7.4) to visualize the DNA.

Slides were viewed with a Nikon Microphot-FX (Nikon, Inc, Melville, N.Y.) microscope. Images were recorded with a Zeiss AxioCam MR camera (Carl Zeiss Vision GmbH, Germany). Minor contrast and gamma adjustments of the images were made with Photoshop software.

### Parasite count

Parasite counts standardized per 200 leukocytes were obtained from thick/thin blood films. The number of parasites per microliter of blood was calculated by assuming an average white blood cell count of 10,000/μl in children.

### Cytokine determination

Plasma and serum samples from children ≤5 years of age (n = 21) were analysed in duplicate both pre-and post-treatment to assess individual cytokine changes. ELISA kits were used to measure IL-10, IL-6, TNF, IL-2, IFN-γ (Pierce Biotechnology, Inc., Rockford, IL), TGF-β, and CRP (US Biological, Swampscott, MA) according to manufacturers' protocols. Positive and negative controls were included. Standard curves were determined from serial dilutions of recombinant human cytokines included in each test kit.

### ANA level determination

Serum samples from children ≤5 years of age (n = 21) were analysed evaluated by the ELISA using an assay kit for all antinuclear antibodies (ANA) as well as a kit specific for IgG ANA to dsDNA, according to the manufacturer's instructions (Wampole Laboratories, Princeton, N.J.). Both pre-and post-treatment samples were analysed to assess changes in individual patient general and anti-dsDNA ANA levels. Additionally, blood obtained during the season of low parasite transmission [46] from age-matched children (n = 18) that were slide negative for detectable trophozoites or gametocytes was evaluated for general ANA. Scoring of ANA levels was based on ANA kit index values: negative ≤ 0.90; equivocal 0.91 – 1.09; positive ≥ 1.10.

### Statistical analysis

Sigma Plot was used to analyse data and assemble graphs. Independent cytokine and ANA values for pre- (n = 21) and post-treatment (n = 21) samples, and anomalies associated with NETs and cytokine induced increased fragility of lymphocytes and neutrophils, as noted on peripheral slide evaluation by leukocyte differential analysis were tested for equality of variances by Levenne's test and equality of means by Student's t test (2-tailed). Results were expressed at a 95% confidence level as the mean ± standard deviation. Cramer's V test was used for measures of nominal association, as might be found in aggregation phenomena and/or the presence of extracellular fibers in fibrinoid complexes. The results of the Cramer's V tests were evaluated according to the following criteria: <0.10 = no relationship, 0.10 – <0.20 = weak association, 0.20 – <0.25 = moderate association, 0.25 – <0.30 = moderately strong association, 0.30 – <0.35 = strong association, 0.35 – <0.40 = very strong association, 0.40 – <0.45 extremely strong relationship or the two variables are measuring the same concept, 0.45 – <0.99 = two variables probably measuring the same concept, 1.00 = perfect relationship; independent variables will predict the dependent variable.

## Results

### Peripheral slide analysis – leukocyte differential counts

Blood samples from 21 children five years of age and younger diagnosed with clinically uncomplicated slide-positive *P. falciparum *malaria were obtained before and seven days after treatment with SP during the season of high transmission. Peripheral slide analysis of Giemsa-stained thick and thin blood films revealed the children had parasitaemias ranging from 2,000/mL to 90,000/mL (mean = 30,738) before SP treatment and negligible trophozoite levels seven days after SP treatment in all but one patient (who had <250 trophozoites/mL). Gametocytes were present in 10% (2/21) of the pre-treatment and 32% (6/19) of the post treatment patients, consistent with the inactivity of SP as a gametocidal drug (data not shown). Seven (34%) of the 21 children presented with hepatomegaly (2 cm in 5 children; 4 cm in 2 children). Two of the children with 2 cm hepatomegaly also presented with 3 cm splenomegaly. No statistical correlation was found between cytokine levels and the presence of gametocytes, hepatomegaly, and/or splenomegaly.

**Figure 1:**
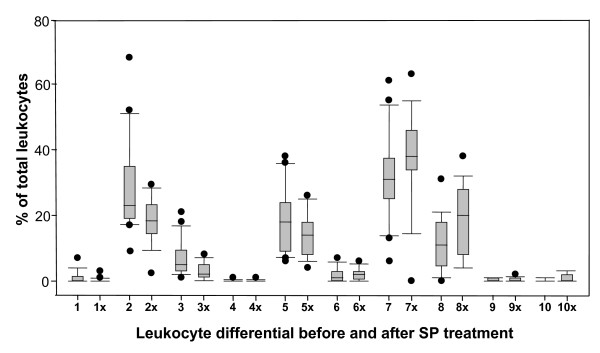
**Leukocyte differential counts before and seven days after SP treatment**. One hundred leukocytes on fingerprick peripheral blood smear slides for each child made before and seven days after SP treatment (x) were classified into one of ten groups. The percentage of each group was calculated. Box plots indicating the 25^th^, 50^th^, and 75^th ^percentile (solid lines from bottom to top of box, respectively), the 90th and 10^th ^percentile values (high and low error bars respectively), and high and low outlier points are shown for each of the following leukocyte classes: 1) metamyelocytes, 2) segmented neutrophils, 3) bands, 4) hypersegmented neutrophils, 5) NETs, 6) monocytes, 7) lymphocytes, 8) smudge forms, 9) eosinophils, and 10) basophils.

Percentages (number per 100 leukocytes counted) of normal and abnormal lymphocytes and neutrophils were determined by a leukocyte differential count before and seven days after initiation of SP treatment with special attention to the presence of NETs (Figure 1). The post-treatment differential analysis was based on slides from 19 of 21 children, because two post treatment slides were not available for analysis in the U.S. Haematologic values were compared to a normal differential reference range [50]. Remarkably, monocyte involvement was low in the children both pretreatment (mean of 1.21% ± 2, range of 1-5/100), in 29% (6/21) of the children, and post treatment (2.0% ± 1.8, 1-6/100), in 68% (13/19) of the children. Monocyte levels did not change significantly after treatment (P < 0.105). Levels of eosinophils and basophils were normal in the children both before and after treatment.

**Figure 2:**
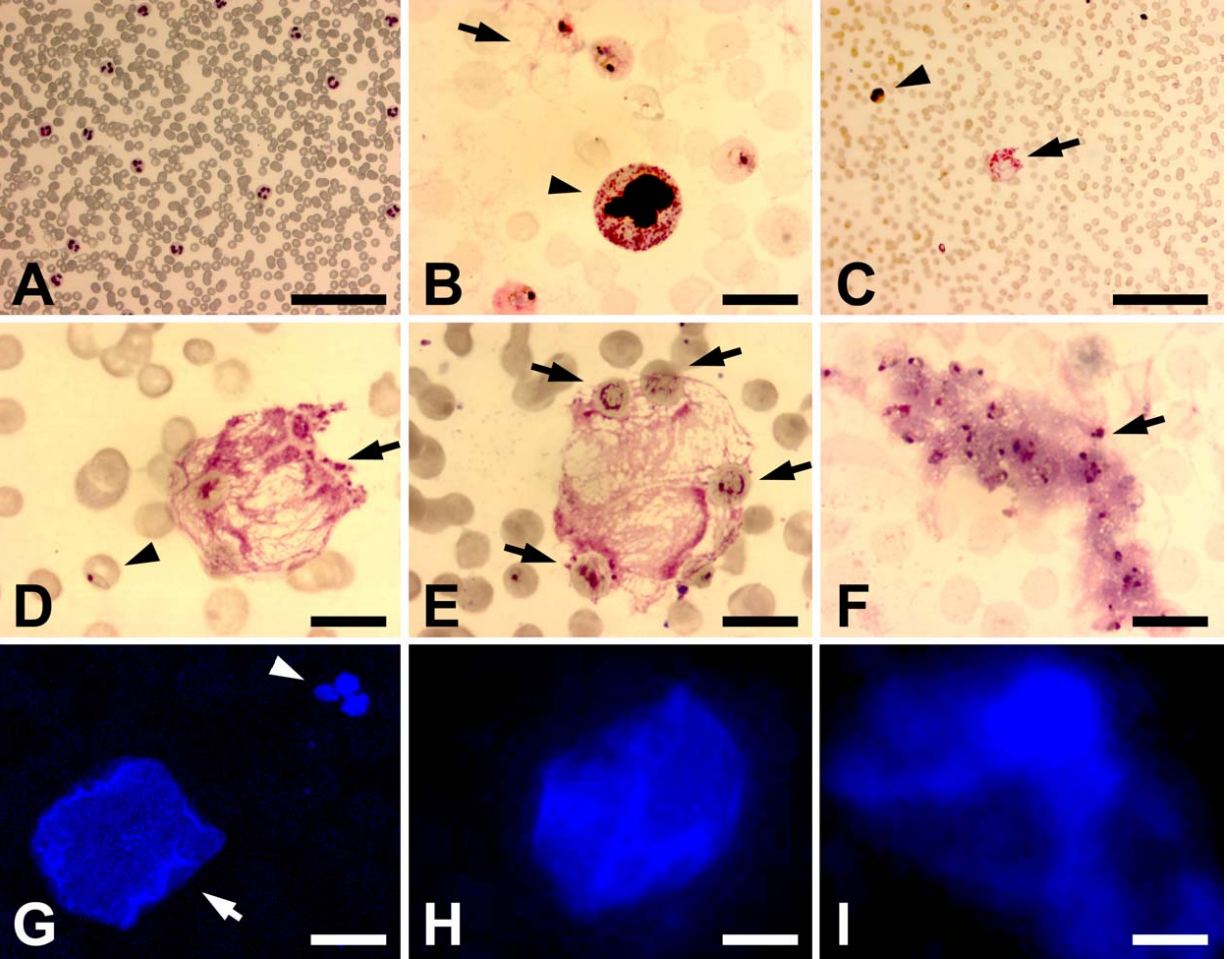
**Giemsa- and DAPI-stained peripheral fingerprick blood smears from children 5 years old and younger presenting with uncomplicated malaria**. (A) low magnification field of a control smear from a child presenting with fever but no detectable *P. falciparum*; (B) field showing neutrophil exhibiting toxic granulation and lack of a distinct nuclear membrane (arrowhead)and fibrous material (arrow); (C) field showing NET (arrow) and *P. falciparum*-infected erythrocyte with a young trophozoite (arrowhead); (D) higher magnification image of field from (C); (E) Fibrous aggregate interpreted to be a NET containing parasitized erythrocytes (arrows); (F) Fibrous aggregate interpreted to be a NET sequestering parasitized erythrocytes; (G) DAPI-stained aggregate resembling fibrous structures in D and E (arrowhead) and normal neutrophil (arrow); (H) DAPI-stained aggregate resembling fibrous structures in D and E; and (I) DAPI-stained aggregate resembling fibrous structure in F. Bars: A, C 100 μm; B, D-F, 10 μm.

Lymphocytes were observed in all (21/21) the children pretreatment (31.7% ± 13, range of 6-61/100), and most (17/19) children post treatment (36% ± 16, range of 0-63/100), with no significant change overall between pre- and post-treatment (P < 0.359). Lymphocyte fragility was evident as leukemoid bare nuclei in smudge cells [51] to varying degrees in 95% (20/21) of the children pre-treatment (10.6% ± 8.2, range of 1-21/100). The mean increase to 18.9% ± 10.7 (range of 4-28/100) in 100% (19/19) of the children post treatment was significant (P < 0.012). Stained control smears from children presenting with fever but lacking evidence of *P. falciparum *on the slides exhibited no evidence of fragile leukocytes (Figure 2A).

Neutrophils dominated the pre-treatment leukocyte population with segmented neutrophils (29.3% ± 14.4, range of 9-68/100) in 100% (21/21) of the children pre-treatment and (18.4% ± 6.6, range of 2-28/100) in 100% (19/19) of the children post treatment; the post-treatment decrease in the mean number of segmented neutrophils was significant (P < 0.005). Hypersegmented neutrophils were observed in only one child (1/21). Immature neutrophils were found as metamyelocytes (1.3% ± 1.9, range of 1-7/100) in 43% (9/21) of the children pre-treatment and in 11% (2/19) of the children post-treatment (0.2% ± 0.7, range of 0-3/100) and as bands (7.2% ± 5.3, range of 1-21/100) in 95% (20/21) of the children pre-treatment and in 84% (16/19) of the children post-treatment (2.7% ± 2.5, range of 1-8/100). The post-treatment drop in bands was significant (P < 0.002), but the drop in metamyelocytes was only borderline significant (P < 0.053). Neutrophils from 14 (67%) of the children exhibited toxic granulation (Figure 2B).

### Neutrophil extracellular traps

All the children exhibited evidence of aggregates of circulating net-like or stranded material with adhered parasitized erythrocytes and trophozoites (Figure 2C–F). The net-like or stranded material stained with DAPI (Figure 2G–I), confirming it contains DNA. The status of the neutrophils and presence of DNA in the circulating aggregates supports the conclusion that these aggregates are NETS. This evidence for NET formation was found in 86% (18/21) of the children pretreatment (18.5% ± 9.9, range of 6-38/100) and in 100% (19/19) of the children post treatment (14.5% ± 6.4, range of 4-26/100). The slight decrease in the mean number of NETs post treatment was not significant (P < 0.155).

### Antinuclear antibodies

**Figure 3:**
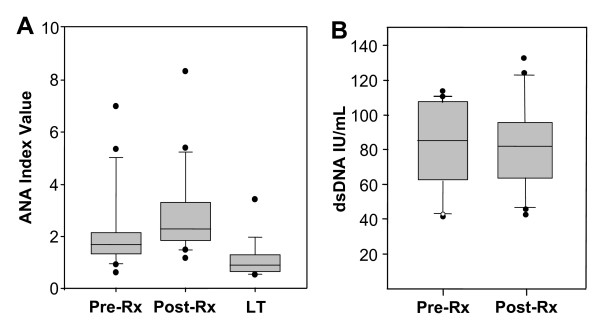
**Anti-nuclear antibody (ANA) and anti-dsDNA levels in 21 falciparum-infected children before and seven days after SP treatment and in age-matched uninfected children**. Samples from the infected children (A and B, Pre-Rx and Post-Rx) were collected during the season of high transmission. The Pre-Rx samples in (A) and (B) were collected before SP treatment. The Post-Rx samples in (A) and (B) were collected seven days after SP treatment. Samples from children exhibiting no evidence of falciparum infection were collected during a season of low transmission (A, LT). ANA Index values were calculated according to assay kit instructions and interpreted as negative, ≤0.90; equivocal, 0.91-1.09; and positive, ≥1.10. Anti-dsDNA levels were calculated according to assay kit instructions and interpreted as negative <25 IU/ml, borderline positive: 25-30 IU/ml, positive: 60-200 IU/ml, and high positive: >200 IU/ml.

To determine whether the presence of DNA in the circulating NETs stimulated an immune response in the infected children, levels of ANA in the samples from the 21 falciparum-infected children were measured before and after SP treatment. ANA values determined for the children infected with malaria were compared to ANA levels in a control group of age-matched children obtained during the season of low transmission that were slide negative for detectable levels of trophozoites or gametocytes. Significantly elevated ANA levels were found in 86% (18/21) of the falciparum-infected children pre-treatment and in 100% (21/21) of the post-treatment serum samples (Figure 3A), but in only 33% (6/18) children of a control group of children whose blood was collected in a season of low transmission (Figure 3). Levels of ANA reactive with dsDNA that are predictive of autoimmunity (>60 IU/ml) were found in 81% (17/21) of the falciparum infected children pre-treatment and post-treatment (Figure 3B).

### Cytokine profiles

**Figure 4:**
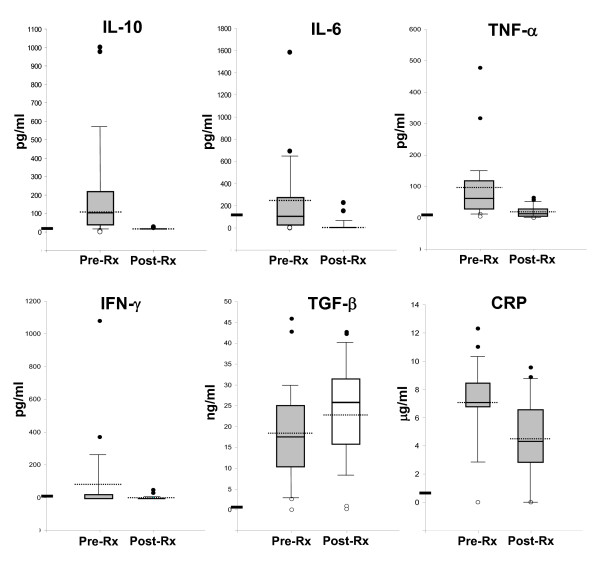
**Levels of IL-10, IL-6, TNF, IFN-γ, TGF-β, and CRP in plasma or serum drawn from 21 children ≤ 5 years old infected with *P. falciparum *before (Pre-Rx) and 7 days after treatment with SP (Rx)**. Individual values (n = 21) are represented as box plots indicating the 25^th^, 50^th^, and 75^th ^percentile (solid lines from bottom to top of box), the 90th and 10^th ^percentile values (high and low error bars respectively), and the mean value (dotted line). Closed circles represent the two highest values (above 90^th ^percentile). Open circles represent the two lowest values (below 10^th ^percentile; two open circles are superimposed in the plots where only one open circle is apparent). Horizontal bars on x-axes represent the upper limit of the range for normal healthy individuals determined with each assay kit by the manufacturer.

Pre- and post-SP treatment plasma samples from all 21 children also were evaluated by ELISA for levels of various cytokines: IL-10, a cytokine directed toward increased Th2 activity; IL-6, a cytokine associated with an acute immune response; TNF a multifunctional cytokine; IL-2, a cytokine associated with T-cell clonal expansion; IFN-γ, a NK- or Th1 cell-mediated adaptive immune response cytokine; TGF-β, a cytokine with immunoregulatory activity; and CRP, an acute phase complement cascade-activating protein. Mean pre-SP treatment levels of all cytokines tested except IL-2 were high compared to healthy US adult standard ranges (Figure 4). Levels of IL-2 found in all pre- and post-treatment samples were below the range of detection in the ELISA assay.

Decreased parasitaemia due to SP treatment correlated with significant decreases in the mean (and median) levels of IL-10 (~30-fold decrease in mean, p = 0.000), IL-6 (~30-fold decrease in mean, p < 0.001), TNF-a (~5-fold decrease in mean, p < 0.006), and IFN-g (~20-fold decrease in mean, p < 0.004) (Figure 3). The dramatic drops in these cytokine levels strongly suggest the pre-SP treatment levels were elevated due to effects associated with *P. falciparum *trophozoite parasite infection, or cytokine inhibition by SP. SP treatment had no significant effect on the post-treatment levels of either TGF-b (p < 0.607) or CRP (p < 0.023) after seven days (Figure 3).

IL-6 levels strongly correlated with the erythrocyte Rouleaux aggregation phenomenon (Cramer's V = .36, p = .104, data not shown) and very strongly correlated with the presence of extracellular fibers (Cramer's V = 0.6, p = .006), which appeared to coat circulating erythrocytes and sequester free-circulating parasites (Figure 2G–I). Extracellular fibers were observed in 13 (62%) of these children, many of whom demonstrated appliqué forms (Figure 2H) of advanced stage *P. falciparum *trophozoites [52].

Further analysis of the pre-SP treatment samples revealed that grouping the samples according to child age revealed significant differences (p = 0.078) in mean levels of only one of the cytokines tested IFN-γ (group data not shown separately). The ten children under 24 months old had a mean IFN-γ level of 190 pg/ml (SD ± 338 pg/ml), with undetectable levels in only two of those children (25% of children under 24 months old), whereas the eleven children 24 to 60 months old had a mean IFN-γ level of 0.47 pg/ml (SD ± 1.1 pg/ml), with undetectable levels in eight of those children (73% of the children over 24 months old). Analysis of individual pre-SP treatment samples revealed that one child, a one-year old female, had the highest levels of IL-6 and TNF (highest of the two outlier points in both Figure 4 box plots), the next to highest level of IL-10 (the second highest outlier point in the Figure 4, IL-10 box plot), and the next to highest IFN-γ (the second highest outlier point in the Figure 4, IFN-γ box plot).

**Figure 5:**
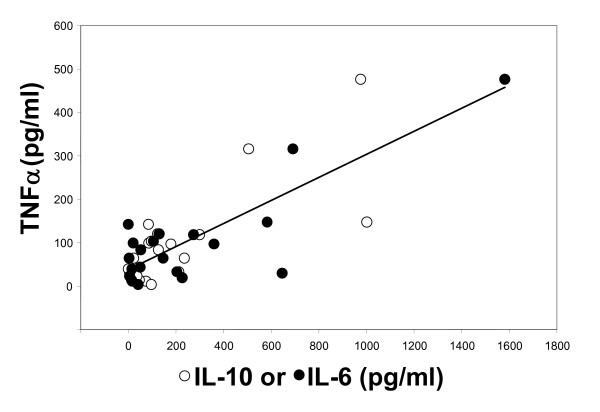
**Triad relationship of TNF, IL-10, and IL-6 levels**. Relationships of TNF with IL-10 (open circles) and with IL-6 (closed circles) are plotted for each of the 21 patients. The additive combinatorial effect of elevated IL-6 and IL-10 cytokine levels on TNF is significant (without random sampling error). Line, multivariate regression line (r^2 ^= 0.726).

**Figure 6:**
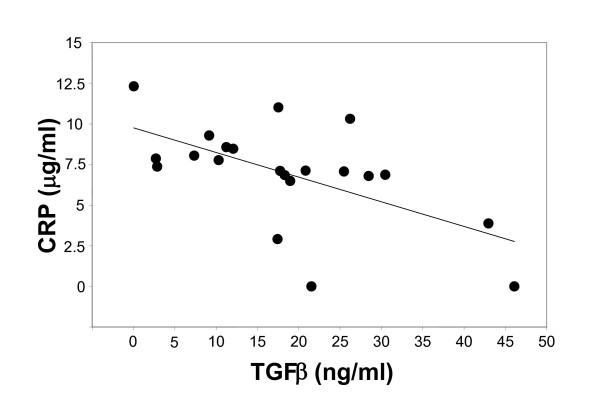
**Relationship of CRP and TGF-β levels in 21 falciparum-infected children before SP treatment**. CRP levels exhibits a modest inverse relationship with TGF-β levels. Line, regression line (r^2 ^= 0.353).

Relating multiple variables in the pre-SP-treatment samples revealed a triad correlation only for levels of IL-6, IL-10, and TNF (Figure 5). The effect of IL-6, while controlling for IL-10, on TNF is statistically significant (virtually without random sampling error; p = 0.009, r = 0.852, r^2 ^= 0.726). Relating TGF-β levels to CRP levels revealed a moderately strong inverse correlation (p = 0.005, r = 0.6, r^2 ^= 0.353) (Figure 6).

**Figure 7:**
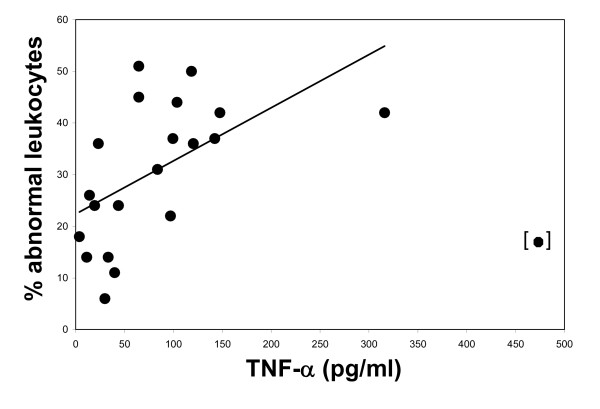
**Relationship between the presence of NETs and elevated TNF levels**. Levels of abnormal leukocytes exhibit a positive relationship with elevated TNF levels. Line, regression line (r^2 ^= 0.512).

Elevated TNF-a levels which did not exceed 324 pg/ml (20/21 children) correlated with NET formation (r^2 ^= 0.512) quantified by the leukocyte differential percentage of NETs and smudge forms determined by peripheral slide analysis (Figure 7). Analysis of individual pre-SP treatment samples revealed that the one year-old female described earlier generated the single outlier point.

## Discussion

Mechanisms underlying malaria-associated immune hyporesponsiveness and pathogenesis remain unclear [4]. This investigation relates leukocyte differential counts, circulating NETs, cytokine profiles, and ANA levels to help elucidate these mechanisms in falciparum malaria pathogenesis in young children residing in sub-Saharan African pockets of endemicity. For this investigation, World Health Organization-trained drug sensitivity testing team members [46] prepared fingerprick peripheral blood smears and obtained blood samples before and seven days after treatment with sulfadoxine-pyrimethamine (SP) from 21 Nigerian northern plateau children five years old and younger diagnosed with clinically-uncomplicated, slide-positive falciparum malaria.

The peripheral slide analysis revealed that all 21 children exhibited some degree of leukocyte pathology including abnormal neutrophils. In the innate immune response, neutrophils can use phagocytosis to kill microbes intracellularly or NET formation to kill microbes extracellularly. To form a NET, the nucleus of an activated neutrophil loses its classic lobular appearance as the nuclear membrane dissolves mixing chromatin with cytoplasmic granules. The neutrophil plasma membrane then ruptures, releasing the chromatin and adherent granule proteins as a coherent NET or in some cases as less organized extracellular fibers [53]. NETs composed of a chromatin scaffold decorated with granule proteins have been shown to kill gram positive and gram negative bacteria as well as some fungi [37,38,40,42,53,54]. In this investigation, various stages of NET formation with bound falciparum parasites were found on the peripheral blood smear slides from the falciparum-infected children. Evidence for NET circulation and involvement in the immune response to falciparum infection has not been previously reported. It remains to be determined whether NET association leads to falciparum parasite destruction.

In addition to possibly killing the parasites, release of neutrophil self-DNA in the NETs found in this investigation could induce autoimmunity, which in turn may contribute to acute severe pathogenesis in young children. In systemic lupus erythematosus, for example, the adjuvant activity of released self-DNA contributes to production of ANA either through an acute response involving complement-fixing antibodies against DNA or nucleoprotein-DNA complexes [19,23,55] or through a Th2-mediated IgG-induced humoral response [55-57]. General ANA levels, as well as IgG anti-dsDNA ANA diagnostic of an autoimmune response, were found in samples collected during the season of high malaria transmission from all the children in this study (86% pre-treatment and 100% post-treatment, 81% pre-treatment and 81% post-treatment; general ANA versus specific IgG dsDNA ANA, respectively). Autoimmune reactions to self-DNA from neutrophil shadow cells of Gumprecht and lymphocyte smudge cells contribute to certain noncancerous leukemias [50,51]. The elevated number of metamyelocytes found in some of the falciparum-infected children in this study may reflect autoimmune activity.

Once produced, ANA may contribute to malarial pathogenesis through formation of immune complexes that induce renal anomalies and hyperuraemia [58-60] or impair leukocyte function [59]. Moreover, NET adherence to parasitized and nonparasitized erythrocytes, as found in this investigation, could generate a break in tolerance and induce a carrier effect anti-erythrocyte Th1 cell-mediated activity leading to severe anaemia [31,61]. Although all the children in this study had a packed red cell volume greater than 25% and, therefore, were not classified as anaemic [47], the presence of burr cells, acanthocytes, helmet cells, and schistocytes on the peripheral blood slides depicted a predisposition to anaemia. As the disease progresses, anti-erythrocyte activity could cause erythrocyte sequestration contributing to development of the microvascular occlusion associated with fatal cerebral malaria [26].

The elevated TNF levels found in this study were coincident with NETs. It is possible that the distinct cytokine profile (the high TNF/Il-6/Il-10 triad relationship with high levels of persistent TGF-β and CRP in the absence of Il-2) and the consistent presence of ANAs found here may represent the NET immunologic response to malaria in this sub-Saharan pocket of endemicity [62].

A moderately strong inverse correlation between TGF-β and CRP was found before and after treatment in this investigation. TGF-β-treated neutrophils produce reactive oxygen species (ROS), which are necessary for NET formation [40], while CRP inhibits neutrophil chemotaxis and production of ROS [63]. CRP participates in opsonization and increased clearance of damaged cells, including opsonized erythrocytes and/or damaged leukocytes, by the classical pathway [20,22,64]. In children with low CRP levels, lack of such protection may permit a lack of clearance of debris and a progression from uncomplicated to severe malaria. Further studies are needed to determine the extent of the inverse relationship between TGF-β and CRP in other age groups within this population to determine whether there is a critical ratio threshold where the balance between the normal activities of TGF-β [65] and CRP [63,66] is lost. In the future, TGF-β activity will be confirmed by measuring levels of IgA resulting from immunoglobulin class-switch.

NET formation and ANA production in older individuals with more adapted immune systems in a population chronically exposed through high seasonal malaria transmission could produce a very different outcome. In the adult population over time, the novel presentation of the parasites captured by NETs could induce a carrier-hapten type of humoral and cellular immune induction similar to that produced by the adjuvant effect of certain bacterial and parasite DNAs [67]. Self-DNA interaction with Toll-like receptor 9 (TLR9), which stimulates IL-12-IFN-γ-dependent activation of Th1 cells, could stimulate immune response not only to the DNA complexes but also to the captured falciparum parasites. Such an adaptive mechanism of protection is consistent with the observation that elevated IL-12 levels are associated with less severe malaria in adults [33]. Cross reacting activation of Th1 and Th2 mechanisms through toll-like receptors has been associated with CpG adjuvant activity [67].

Transition of infected children from autoimmune pathogenesis to autoimmune protection may depend on developing the ability to mount a T cell independent pathogen associated molecular pattern (PAMP) response. The lack of detectable IL-2 in the children with uncomplicated falciparum malaria in this study could be because it is bound to a soluble IL-2 receptor and missed by the assay [62]. Alternatively, it may represent a lack of Th1 and Th2 clonal expansion, with a shift to NET-induced activation [68] through Toll-like receptors. If NET DNA binds to TLR9 and the ANA that is present in the children binds to IgG receptors, dendritic cells may cross present both major histocompatibility complexes I and II, thereby enhancing both humoral- and cell-mediated immune responses. As the ability of the child to mount a PAMP immune response develops, this autoimmune activity may become protective against the parasite by activating T cytotoxic cell activity [44]. Development of NET-induced PAMP activity therefore could explain the paradox that high IFN-γ levels are associated with severe malaria in young children who have low IL-10 levels [5] but also associated with decreased malarial morbidity in adults who have elevated IL-12 levels associated with an adaptive Th1 [33] and Th2 response [67].

Ironically, development of ANA, as found in the children assessed in this investigation, may bind well enough to CpG to neutralize its adjuvant activity. This could contribute to immune hyporesponsiveness [4] and affect the outcome in CpG adjuvant-based vaccines [43].

## Conclusion

This investigation of blood samples from 21 children in a mesoendemic region of sub-Saharan Africa with uncomplicated falciparum malaria provides evidence for the formation of circulating NETs that can capture the falciparum parasites and stimulate production of ANA. In conjunction with leukocyte differential counts and cytokine profiles, the presence of the NETs and ANA supports the premise that falciparum-induced autoimmune activity contributes to pathogenesis in the children, but may provide a TNF-based mechanism of protection through recurrent PAMP activation during chronic exposure over time [44].

## Authors' contributions

VB, MB and TK designed the study. VB, GI, NM, SP, MO, SS, DE, DI, BA, MB, KF, and RF obtained and prepared the samples. VB performed the research analyses. VB, PT, KR, and TK analysed the data. VB, KR, and TK wrote the paper. All authors read and approved the final manuscript.
